# Diaphragm Muscle Weakness in an Experimental Porcine Intensive Care Unit Model

**DOI:** 10.1371/journal.pone.0020558

**Published:** 2011-06-15

**Authors:** Julien Ochala, Guillaume Renaud, Monica Llano Diez, Varuna C. Banduseela, Sudhakar Aare, Karsten Ahlbeck, Peter J. Radell, Lars I. Eriksson, Lars Larsson

**Affiliations:** 1 Department of Neuroscience, Uppsala University, Uppsala, Sweden; 2 Section of Anesthesiology and Intensive Care Medicine, Karolinska Institute, Stockholm, Sweden; 3 Department of Biobehavioral Health, The Pennsylvania State University, University Park, Pennsylvania, United States of America; Rutgers University, United States of America

## Abstract

In critically ill patients, mechanisms underlying diaphragm muscle remodeling and resultant dysfunction contributing to weaning failure remain unclear. Ventilator-induced modifications as well as sepsis and administration of pharmacological agents such as corticosteroids and neuromuscular blocking agents may be involved. Thus, the objective of the present study was to examine how sepsis, systemic corticosteroid treatment (CS) and neuromuscular blocking agent administration (NMBA) aggravate ventilator-related diaphragm cell and molecular dysfunction in the intensive care unit. Piglets were exposed to different combinations of mechanical ventilation and sedation, endotoxin-induced sepsis, CS and NMBA for five days and compared with sham-operated control animals. On day 5, diaphragm muscle fibre structure (myosin heavy chain isoform proportion, cross-sectional area and contractile protein content) did not differ from controls in any of the mechanically ventilated animals. However, a decrease in single fibre maximal force normalized to cross-sectional area (specific force) was observed in all experimental piglets. Therefore, exposure to mechanical ventilation and sedation for five days has a key negative impact on diaphragm contractile function despite a preservation of muscle structure. Post-translational modifications of contractile proteins are forwarded as one probable underlying mechanism. Unexpectedly, sepsis, CS or NMBA have no significant additive effects, suggesting that mechanical ventilation and sedation are the triggering factors leading to diaphragm weakness in the intensive care unit.

## Introduction

Approximately 40% of intensive care unit (ICU) patients are mechanically ventilated for a median duration of five to seven days [1] and problems weaning from the ventilator are observed in 20 to 30% of these patients [2]. A dramatic reduction in the maximal transdiaphragmatic pressure upon supra-maximal phrenic nerve stimulation has been reported in various animal models in response to mechanical ventilation [3], [4], [5]. This decrease in force-generating capacity is not related to modifications in the lung volume, abdominal compliance [5] or phrenic nerve function [4], suggesting a primary diaphragm muscle dysfunction. Diaphragm muscle biopsies from rodent models and humans have revealed cell changes consisting of severe atrophy [6] and disrupted contractility, i.e., loss of maximal force production normalized to cross-sectional area (CSA) [7]. These cell modifications are thought to be the result of reduced contents in contractile proteins such as myosin and actin due to a decreased synthesis rate [8] and increased rate of degradation [9], [10].

Diaphragm cell dysfunction associated with prolonged mechanical ventilation may also be secondary to other factors in the ICU setting, such as sepsis, systemic corticosteroid (CS) treatment and administration of neuromuscular blocking agents (NMBA), but the specific role of these conditions in combination with mechanical ventilation remains in question [11], [12], [13]. The aim of this project was therefore to study the effects of sepsis, CS and NMBA in different combinations on diaphragm muscle structure and function at the cell and molecular levels. For this purpose, we have used a porcine ICU model [4], [14] where all piglets are sedated and mechanically ventilated for five days. All results from the experimental animals were compared with sham-operated control animals. According to the literature, sepsis [15], administration of CS [16] or denervation [17] alone, are sufficient to provoke respiratory muscle cell modifications such as atrophy and a decrease in maximal force production normalized to CSA. In consequence, we hypothesized that, when the above ICU setting factors are associated to mechanical ventilation and sedation, separately or in combination, they have important additive negative effects on diaphragm muscle function. To the best of our knowledge, no other long-term studies using mechanical ventilation together with sepsis, CS and NMBA have previously been performed.

## Materials and Methods

### Animals

A detailed description of animals has been given elsewhere [14]. Briefly, 22 female domestic piglets (23–30 kg body weight) were included (Table 1). Animals were sedated with medetomidine (Dormitor vet 1 mg/ml, Orion Pharma AB, Stockholm, Sweden) and zolazepam (Zoletil 250, Reading, Carros, France). After arrival in the laboratory and preparing an iv line, 100 mg of ketamine (Ketaminol vet 50 mg/ml, Intervet, Boxmeer, Netherlands) was administered. During the 5-day study period, the animals were sedated using isoflurane inhalation (Abbott Laboratories, 0.8–1.3% end-tidal concentration) supplemented by intravenous bolus doses of morphine and ketamine as needed. After induction, the trachea was cannulated via a midline incision and a trachestomy tube was placed distally to the cricothyroid cartilage. All animals were then mechanically ventilated using volume-controlled ventilation (Servo 300; Siemens Elema) with an initial FiO_2_ of 0.21–0.30, an inspired tidal volume of 10 ml·kg^−1^and a respiratory rate of 20 breaths per min, with an I:E ratio of 1:2 and inspiratory rise time of 5–10%. During the remaining study period, the settings were adjusted carefully to maintain arterial oxygen and carbon dioxide tensions within normal limits and to avoid high airway pressures and risk of barotrauma. Sedation was titrated to promote ventilator synchrony and inhibit spontaneous breathing activity. Arterial and central venous catheters were placed in the common carotid artery and the internal jugular vein via a separate incision on the neck and under antiseptic conditions. Arterial and central venous blood pressures, airway pressures and volumes as well as dynamic compliance, rectal temperature and standard three-lead ECG were continuously monitored. For assessment of the hemodynamic conditions, a Swan-Ganz standard thermodilution pulmonary artery catheter was inserted through the internal jugular vein catheter and advanced until the characteristic pulmonary artery pressure curve was registered. Excessive heat loss was avoided by covering the animals when necessary with a warming blanket. A urinary catheter was placed in the urinary bladder for continuous monitoring of urinary output. Arterial acid-base balance, oxygen and carbon dioxide tensions, electrolytes and blood glucose concentration were monitored using ABL 2 and OSM2 analyzers (Radiometer) and a Glucometer (Bayer Health Care). A continuous infusion of Ringer's acetate at 2000–4000 ml.day^−1^ was given for fluid replacement together with a continuous infusion of buffered glucose 2.5 mg·ml^−1^ adjusting the infusion rate to maintain tight control of arterial blood glucose levels between 4–8 mM, and to assure a urinary output of 25–50 ml·h^−1^ throughout the study period. Enteral feeding was not considered practical in this model due to the relatively deep sedation, in some cases paralysis and a small laparotomy used in some study protocols. Previous attempts to provide additional calories with parenteral nutrition solutions resulted in fat vacuolization in muscle cells [4]. All wounds were carefully cleaned and closed by standard sutures to avoid unwanted contamination. During the whole study period, from day 2 each animal received prophylactic streptomycin 750 mg·d^−1^ and benzylpenicillin 600 mg·d^−1^ (Streptocillin Vet, Boeringer-Ingelheim). The animals were clinically monitored and cared for continuously for the entire study period by one of the authors or by a nurse anesthetist experienced in the care of piglets and knowledgeable about the study protocol. Routine care included evaluation of sedation level, changing position, suctioning, adjusting fluid administration, etc. After initial preparation, animals were allowed to stabilize for one hour. This study protocol was approved by the Karolinska Institutet Ethical Committee on Animal Research (Permit numbers: Dnr N71/98, N54/02 and N75/04).

**Table 1:** Animal treatment: CTL (control), MV (mechanical ventilation), sepsis (endotoxemia was induced by a continuous infusion of Escherichia coli endotoxin), NMBA (neuromuscular blocking agent, i.e., continuous infusion of rocuronium, 25 mg·h^−1^), CS (corticosteroid given as bolus doses of hydrocortisone 50 mg, ×3 daily) and ALL (MV + CS + sepsis + NMBA).

Group	N	Mean weight	Survival	PaO2 (kPa)	Compliance (ml/cm H_2_0)	BE (mmol/L)	PP (cm H_2_0)	Endotoxin	CS	NMBA
CTL	4	26.80 kg	3 hours	–	–	–	–	-	-	-
MV	4	25.90 kg	5 days	18.8	22	3.8	28	-	-	-
Sepsis	4	25.50 kg	5 days	17.2	22	1.8	26	E. coli	-	-
CS	3	26.90 kg	5 days	17.3	26	5.2	23	-	Hydrocortisone	-
NMBA	3	26.00 kg	5 days	21.3	25	3.3	25	-	-	Rocuronium
ALL	4	26.80 kg	5 days	19.8	24	4.3	23	E. coli	Hydrocortisone	Rocuronium

N: number of animals; BE: base excess; PP: peak pressure.

The animals were randomly assigned to one of the six groups: CTL, MV, sepsis, NMBA, CS, and ALL. CTL group refers to sham-operated piglets that served as controls and were mechanically ventilated for 3 hours (Table 1). MV group refers to piglets that were mechanically ventilated for five days. Sepsis group refers to mechanically ventilated piglets in which endotoxemia was induced by a continuous one-hour infusion of Escherichia coli endotoxin, serotype O26:B6 (Sigma Labkemi) initially at 36 µg·kg^−1^·h^−1^ and then titrated to effect, total dose 20–30 µg·kg^−1^. The infusion was started at a low dose and titrated upward until a hemodynamic response occurred consisting of a fall in arterial mean blood pressure ≥30% from baseline, with an increase ≥ 50% in pulmonary artery occlusion pressure from baseline. The infusion was paused if the animals required fluid resuscitation or administration of adrenalin for bradykardia and was then restarted at a lower dose. If the animal required repeated interventions the infusion could be terminated earlier than at one hour, and if tolerated could run for up to four hours. Continuous infusions of inotropic drugs were not used to support the circulation. This regimen typically resulted in a 6 to 12 hour-period of severe circulatory instability and oliguria (urinary output <25 ml·h^−1^). Animals surviving this period of septic shock generally regained circulatory stability during the remaining experimental period. NMBA group refers to mechanically ventilated piglets in which a neuromuscular blocking agent was administered as a continuous infusion of rocuronium (Esmeron, Organon) 25 mg·h^−1^ for 5 days while CS group refers to mechanically ventilated piglets in which a corticosteroid was given as bolus doses of 50 mg of hydrocortisone (Solu-Cortef, Pfizer AB) administered three times daily throughout the experiment. ALL group refers to piglets that were mechanically ventilated for 5 days together with the induced-endotoxemia, corticosteroid administration three times daily and continuous systemic administration of a neuromuscular blocking agent. Animals were euthanized by a lethal injection of pentobarbitone and arterial bleeding to terminate the experiment.

### Muscle Biopsies and Permeabilization of Fibres

Biopsy specimens (2×2 cm) from the left anterior costal diaphragm were obtained after abdominal incision prior to euthanizing the animal at the end point (day 5) of the experiment for all piglets from MV, Sepsis, NMBA, CS, and ALL groups. The piglets from CTL were sacrificed at the start of the experiment. Biopsy specimens were dissected into two parts. One part was frozen in liquid nitrogen-chilled propane and stored at −80°C. The other was placed in relaxing solution at 4°C, and bundles of ∼50 fibres were dissected free and then tied with surgical silk to glass capillary tubes at slightly stretched lengths. The muscle bundles were then treated with skinning solution (relaxing solution containing glycerol; 50:50 v/v) for 24 hours at 4°C, after which they were transferred to −20°C. The muscle bundles were treated with sucrose, a cryo-protectant, within 1–2 weeks for long-term storage [18]. After the sucrose treatment, muscle bundles were detached from the capillary tubes and snap frozen in liquid nitrogen-chilled propane and stored at −160°C.

### Single muscle fibre experimental procedure

On the day of an experiment, a fibre segment 1 to 2 mm long was left exposed to the experimental solution between connectors leading to a force transducer (model 400A, Aurora Scientific) and a lever arm system (model 308B, Aurora Scientific) [19]. The total compliance of the attachment system was carefully checked and remained similar for all the single muscle fibres tested (5±0.5% of the fibre length). The apparatus was mounted on the stage of an inverted microscope (model IX70; Olympus). While the fibre segments were in relaxing solution, the sarcomere length was set to 2.65–2.75 µm by adjusting the overall segment length [20]. The diameter of the fibre segment between the connectors was measured through the microscope at a magnification of ×320 with an image analysis system prior to the mechanical experiments. Fibre depth was measured by recording the vertical displacement of the microscope nosepiece while focusing on the top and bottom surfaces of the fibre. The focusing control of the microscope was used as a micrometer. Fibre cross-sectional area (CSA) was calculated from the diameter and depth, assuming an elliptical circumference, and was corrected for the 20% swelling that is known to occur during skinning [19].

Relaxing and activating solutions contained (in mM) 4 Mg-ATP, 1 free Mg^2+^, 20 imidazole, 7 EGTA, 14.5 creatine phosphate, and KCl to adjust the ionic strength to 180 mM. The pH was adjusted to 7.0. The concentrations of free Ca^2+^ were 10^−9^ M (relaxing solution) and 10^−4.5^ M (activating solution), expressed as pCas (i.e., −log [Ca^2+^]). Apparent stability constants for Ca^2+^-EGTA were corrected for temperature (15°C) and ionic strength (180 mM). The computer program of Fabiato [21] was used to calculate the concentrations of each metal, ligand, and metal-ligand complex.

At 15°C, immediately preceding each activation, the fibre was immersed for 10–20 s in a solution with a reduced Ca^2+^-EGTA buffering capacity. This solution is identical to the relaxing solution except that the EGTA concentration is reduced to 0.5 mM, which results in more rapid attainment of steady force during subsequent activation.

#### Force

This was calculated as the difference between the maximal steady-state isometric force in activating solution and the resting force measured in the same segment while in the relaxing solution. Maximal force production was normalized to CSA (specific force, P_0_/CSA).

#### Apparent rate constant of force redevelopment

Once steady-state maximal isometric force was reached, a slack by 20% of the original fibre length was rapidly introduced (within 1–2 ms) at one end of the fibre, resulting in a rapid reduction of force to near zero. This was followed by a brief period of unloaded shortening (20 ms), after which the preparation was quickly restretched to its original length and the force was recovered to its original steady-state value. As described previously [22], the apparent rate constant of force redevelopment (k_tr_) was estimated by linear transformation of the half-time of force redevelopment (t_1/2_) as follows [23]:
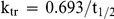



#### Stiffness

Once maximal steady-state isometric force was reached, small-amplitude sinusoidal changes in length (ΔL: ± 0.2% of fibre length) were applied at 500 Hz at one end of the fibre [24]. The resultant force response (ΔF) was measured, and the mean of 20 consecutive readings of ΔL and ΔF was used to determine stiffness. The actual elastic modulus (E_0_) was calculated as the difference between E in activating solutions and resting E measured in the same segment in the relaxing solution. E was determined as follows [25]:
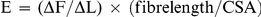



Stiffness was measured at pCa 4.5 in the presence and absence (rigor solution) of ATP.

For contractile measurements, strict acceptance criteria were applied. First, the sarcomere length was checked during the experiments, using a high-speed video analysis system (model 901A HVSL, Aurora Scientific). A muscle fibre was accepted and included in the analyses: (i) if the sarcomere length of a single muscle fibre changed <0.10 µm between relaxation and maximum activation, (ii) if maximal force changed <10% from first to final activation [19].

After the mechanical measurements, each fibre was placed in urea buffer (120 g urea, 38 g thiourea, 70 ml H_2_0, 25 g mixed bed resin, 2.89 g dithiothreitol, 1.51 g Trizma base, 7.5 g SDS, 0.004% bromophenol blue) in a plastic micro centrifuge tube and stored at −80°C.

### Myosin Heavy Chain (MyHC) isoform expression

The MyHC isoform composition of fibres was determined by 6% sodium dodecyl sulfate polyacrylamide gel electrophoresis (SDS-PAGE). Sample loads were kept small (equivalent to ∼0.05 mm of fibre segment) to improve the resolution of the MyHC bands (slow and fast MyHC: type I, IIa, IIx and IIb). Electrophoresis was performed at 120 V for 24 h with a Tris–glycine electrode buffer (pH 8.3) at 15°C (SE 600 vertical slab gel unit, Hoefer Scientific Instruments). The gels were silver-stained and subsequently scanned in a soft laser densitometer (Molecular Dynamics) with a high spatial resolution (50 µm pixel spacing) and 4096 optical density levels.

MyHC isoform expression was also measured from frozen diaphragm muscle samples, i.e., the samples were cut at their greatest girth perpendicular to the longitudinal axis of muscle fibres into 10-µm-thick cross-sections with a cryotome (2800 Frigocut E, Reichert-Jung) at −20°C. The 10-µm cross-sections of each muscle were dissolved in urea buffer and a volume of 4 µl was loaded on 6% SDS-PAGE. The gels were then stained with Coomassie blue (0.5 g brilliant blue, 225 ml MeOH, 225 ml distilled H_2_O and 50 ml acetic acid), as this staining shows high reproducibility and the ability to penetrate the gel and stain all proteins present, i.e., allowing accurate quantitative protein analyses. The gels were subsequently scanned to determine the fibre type proportion.

### Myosin, actin and total protein quantification

To quantify the amount of total protein, cross-sections were dissolved into 100 µl of urea buffer after centrifugation and heating (90°C for 2 minutes). Total protein quantification of the samples was performed using the NanoOrange**®** Protein Quantification Kit (Invitrogen), the fluorescence of the samples was measured using a Plate Chamelen™ Multilabel Platereader (Hidex Oy) and the software MikroWin2000, version 4.33 (Microtek Laborsysteme GmbH). The fluorescence of the samples was related to a standard curve made using bovine serum albumin (Invitrogen) at concentrations ranging from 10 µg·ml^−1^ to 0.1 µg·ml^−1^.

Myosin and actin quantification was determined by 12% SDS-PAGE. Volumes of 5 µl of the samples were loaded along with 5 µl of the standard dilutions. The standard was prepared by pooling sections from control animals. The myofibrillar protein standards were prepared, assuming that actin and myosin contents were 12.5 and 25% of the total protein content, respectively. Linear actin and myosin curves were observed within the 5–200 µg.ml^−1^ range but the calibration curves were not parallel. Myosin and actin contents were normalized to total protein content.

### Measurement of reactive carbonyl derivatives

The reactive carbonyl derivatives of myosin and actin were determined using the Oxy-Blot protein oxidation detection kit (Invitrogen) via 12% SDS-PAGE gels and Western blotting [26]. All the samples were loaded with identical volumes and protein quantities (2.5 µg each). The reactive carbonyl derivatives were quantified using a soft laser densitometer (arbitrary unit).

### Real-time PCR

Quantitative PCR analysis was performed as previously described [14]. Briefly, total RNA (100 ng) was reverse transcribed to cDNA using Qscript cDNA supermix (Quanta Biosciences, USA). cDNA was amplified in triplicate using MyiQ™single color real time PCR detection system (Bio-Rad Laboratories), and used to quantify the mRNA levels for porcine myosin heavy chain type I and IIx, and actin. The thermal cycling conditions include 95°C for 9 minutes, followed by 50 cycles of amplification at 95°C for 15 minutes, followed by 60°C for 1 minute. Each reaction was performed in a 25 µl volume with 0.4 µM of each primer and 0.2 µM probe or SYBR green (1988123, Roche Diagnostics, GmBH). Taq man primers and probes were designed using Primer Express® program (Applied Bio System, Foster). The 18S ribosomal RNA was co-amplified with the target cDNA to serve as an internal standard and allow for correction for differences in starting amounts of total RNA. Primer sequences for porcine myosin heavy chain isoforms and actin are published elsewhere [14].

### Statistical analysis

**Figure 1:**
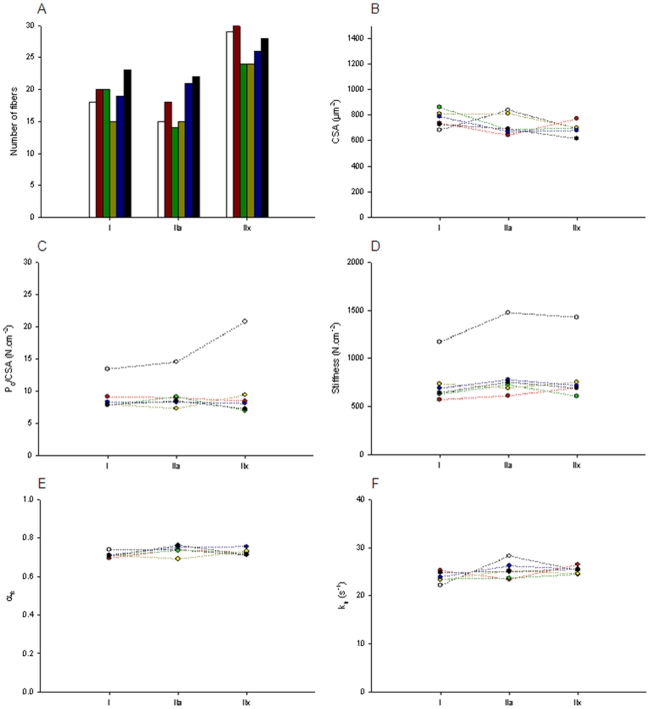
Fibre structure and function. [A] Number of fibres tested. [B] Cross-sectional area (CSA). [C] Maximal force production normalized to CSA (P_0_/CSA). [D] Stiffness (E_0_). [E] Fraction of strongly attached cross-bridges (α_fs_). [F] Apparent rate constant of force redevelopment (k_tr_). Values from CTL (white), MV (red bars), NMBA (green), CS (yellow), sepsis (blue) and ALL groups (black) appear. Data are presented as means.

A total of 383 diaphragm fibres were isolated and tested, i.e., minimum of 15 acceptable fibres per animal (Fig. 1A). For CSA, P_0_/CSA, E_0_, k_tr_ and α_fs_, comparisons were restricted to fibres expressing the type I, IIa and IIx MyHC isoforms. Because a certain amount of fibres was studied for each animal for each treatment, a specific model was used to analyze the data. This model was based on an analysis of variance including the following factors: treatment, MyHC expression and animal (where animal was nested within treatment). The only interaction terms that were judged to be of importance and therefore included were that between treatment group and fibre type. The SAS JMP software was used for the generation of such model. In addition, a one-way ANOVA (with group as the factor) was used for comparisons of MyHC expression, MyHC and actin contents, oxidation and mRNA levels. Sigma Stat software was used. Data are presented as means ± standard error of the means (SEMs).

## Results

### Clinical course

Three animals, all in the endotoxin groups, did not survive the initial phase of the protocol; two animals had near cardiac arrests but were resuscitated and could continue after fluid and bolus doses of adrenalin. Following the changes in hemodynamics used to guide titration of endotoxin, the animals returned to baseline blood pressures which were maintained to the end of the experiments. All endotoxin treated animals developed a significant degree of fever, usually in the range of 39–40.5°C. All animals increased in weight after surgical preparation/inflammation due to fluid retention, but this resolved during the 5 day-protocol and there was no significant change in body weight between day 1 and day 5. Hemoglobin levels fell in all animals due to regular blood sampling, the lowest levels in the range of 60–70 mg L^−1^. Blood glucose and arterial oxygen and carbon dioxide were kept in the normal range, no animal developed more than transient metabolic acidosis and lactate levels were in the normal range.

### MyHC isoform proportion

**Figure 2:**
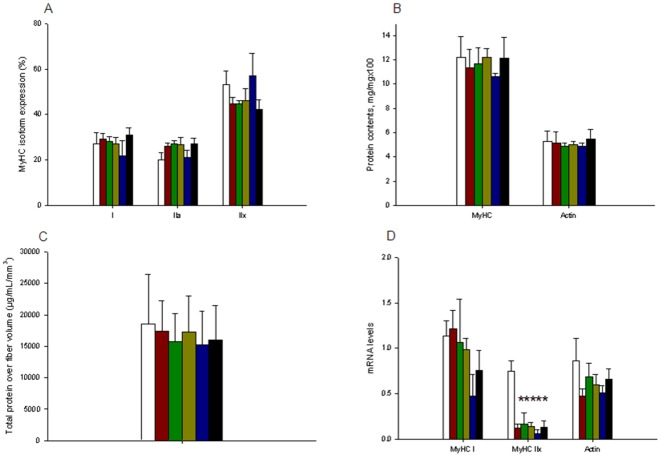
Protein and gene expressions. [A] MyHC isoform composition. [B] Contractile protein content. [C] Total protein over fibre volume. [D] mRNA levels of contractile proteins. Values from sham-operated control piglets (CTL group, white bars) and animals that were mechanically ventilated (MV group, red bars), mechanically ventilated with a neuromuscular blocking agent administration (NMBA group, green bars), mechanically ventilated with corticosteroid administration (CS group, yellow bars), mechanically ventilated with an injection of a endotoxin-induced sepsis (sepsis group, blue bars) and mechanically ventilated with a combination of endotoxin-induced sepsis, CS and NMBA (ALL group, black bars) for five days. Data are presented as means ± SEMs. Asterisk denotes a statistically significant difference compared with CTL (p<0.05).

For all the intervention groups (MV, CS, sepsis, NMBA and ALL), the type I, IIa and IIx MyHC isoform distributions in 10-µm-thick cross-sections were similar to those from the CTL group (Fig. 2A).

### Single muscle fibre experimental procedure

The single muscle fibre preparation allows direct measurements of contractile function in cells with an intact myofilament lattice, but without the confounding effects of nerves, excitation-contraction coupling, fibre architecture and inter-cellular connective tissue. Thus, after permeabilization, diaphragm muscle fibres were isolated from the bundles and mounted for analysis of CSA (at a fixed sarcomere length), specific force (P_0_/CSA), stiffness (E_0_) and apparent rate constant of force redevelopment (k_tr_). A total of 383 fibres were included in the analyses (Fig. 1A). The CSA for all mechanically ventilated piglets (MV, CS, sepsis, NMBA and ALL) was similar to the CTL group (Fig. 1B). On the other hand, specific force (P_0_/CSA) decreased after 5 days for all intervention groups (MV, sepsis, CS, NMBA and ALL), in fibres expressing type I, IIa and IIx MyHC isoforms (p<0.05) (Fig. 1C). Specific force is mainly determined by the number of strongly attached myosin-actin interactions, i.e., cross-bridges, and the force produced by each cross-bridge. An index of the number of strongly attached cross-bridges can be obtained when calculating maximal stiffness (E_0_). As for specific force, E_0_ was reduced after 5 days the mechanically ventilated piglets (MV, sepsis, CS, NMBA and ALL), in fibres expressing type I, IIa and IIx MyHC isoforms (p<0.05) (Fig. 1D). To evaluate whether E_0_ changes are due to modifications in the recruitment of myosin heads, the ratio of E_0_ during maximum Ca^2+^ activation to the one during rigor activation was calculated (α_fs_). In all fibre types, this ratio was unchanged (Fig. 1E).

During maximum Ca^2+^ activation, cross-bridges assume at least two distinct conformations, strongly attached to actin, i.e., force-generating state, and weakly attached or dissociated from actin, i.e., non force-generating state. The force produced by each cross-bridge is mostly dependent on its turnover rate. k_tr_ represents the turnover of cross-bridges between the two states that can be described by two rate constants, f_app_ and g_app_, with f_app_ being the transition from the non-force-generating state to the force-generating state, and g_app_ describing the return to the non-force-generating state. k_tr_ of fibres expressing type I, IIa and IIx MyHC isoforms from all mechanically ventilated piglets (MV, sepsis, CS, NMBA and ALL groups) were similar to those from piglets belonging to the CTL group (Fig. 1F).

### Myosin, actin and total protein contents

Myosin and actin contents normalized to total protein content were preserved for all mechanically ventilated piglets (MV, sepsis, CS, NMBA and ALL groups) when compared with controls (Fig. 2B). There was no preferential loss of these contractile elements. To identify a potential general decline in the protein quantity per muscle fibre unit, the amount of total protein per fibre volume was estimated in a separate number of fibres. This amount was unchanged in all experimental piglets when compared with control animals (Fig. 2C).

### Oxidative damages

**Figure 3:**
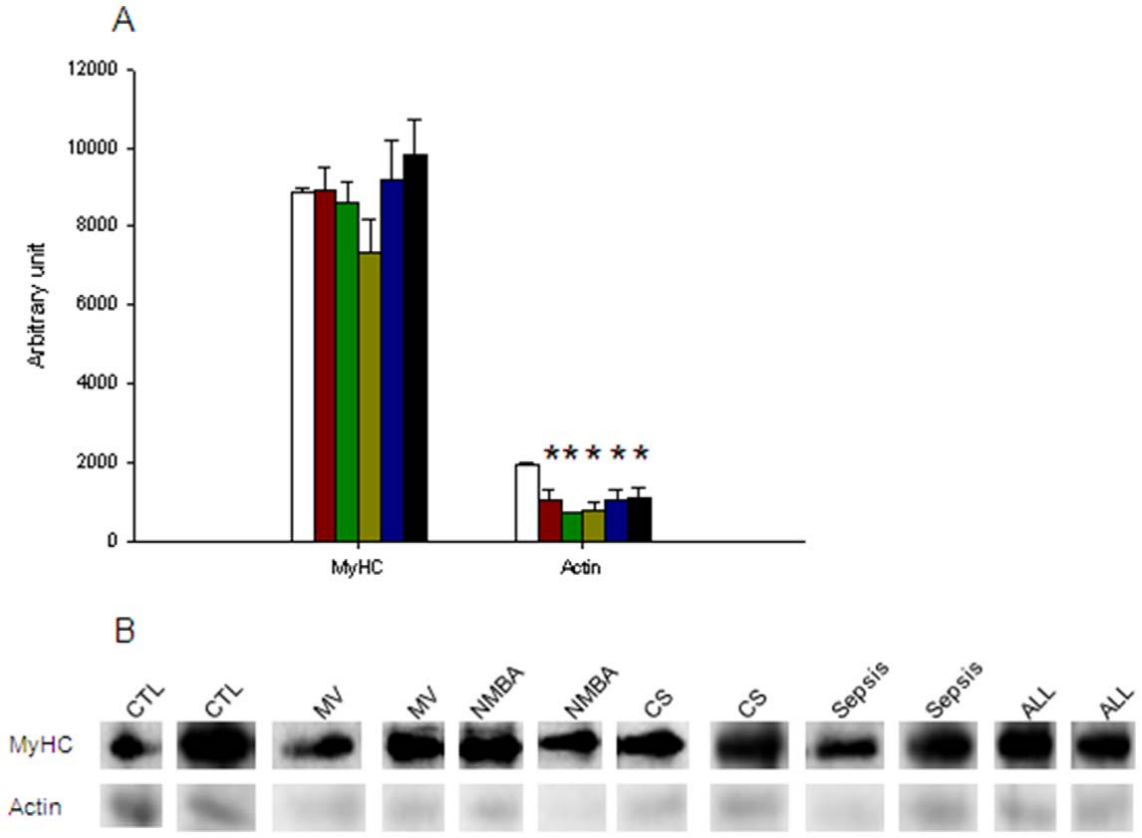
Contractile protein reactive carbonyl derivatives. [A] CTL (white bars), MV (red bars), NMBA (green bars), CS (yellow bars), sepsis (blue bars) and ALL groups (black bars). [B] Typical western blot showing reactive carbonyl derivates for various piglets. Data are presented as means ± SEMs. Asterisk denotes a statistically significant difference compared with CTL (p<0.05).

Oxidative modification of myosin and actin by free radical species and other reactive species usually results in the formation of carbonyl groups into amino acid side chains by a site-specific mechanism. These carbonyl groups or reactive carbonyl derivatives were identified for myosin and actin. Myosin oxidation was found unchanged whereas actin reactive carbonyl derivatives decreased (p<0.05) after 5 days for all intervention groups (MV, sepsis, CS, NMBA and ALL) (Fig. 3).

### MyHC and actin mRNA levels

Real time PCR was used. Even though discrepancies were present between piglets of a same group, type I MyHC isoform and actin mRNA levels were maintained (Fig. 2D). On the other hand, type IIx MyHC isoform mRNA expression was significantly decreased (p<0.05) after 5 days for all mechanically ventilated groups (MV, sepsis, CS, NMBA and ALL).

## Discussion

An increasing number of critically ill ICU patients suffer from severe muscle wasting and impaired diaphragm muscle function, in many cases contributing to delays in weaning from mechanical ventilation. In an attempt to unravel the underlying mechanisms, various experimental animal models have been introduced and given very important information suggesting that prolonged mechanical ventilation plays a negative role [27]. However, these rodent models have mostly been restricted to short durations (up to 1–2 days) and omit other concurrent conditions such as sepsis and common interventions used in modern intensive care (NMBA and CS). Our porcine model considers several of these specific conditions and with a longer duration (five days) [14], [28]. Present results demonstrate that five days of mechanical ventilation and sedation provoke a severe disruption of diaphragm muscle fibre contractility without any major structural remodeling at the cell and protein levels. Surprisingly, sepsis, CS and NMBA, examined separately or in combination, do not add significant negative effects to the ventilator-induced diaphragm changes.

### Ventilator-related modifications

A number of different muscles are involved in respiration and the diaphragm is the dominate inspiratory muscle [29]. To fulfill the continuous heterogeneous functional demand, this particular muscle is composed of both slow (type I MyHC) and fast (type IIa and IIx MyHC) fibres [30]. In the present study, five days of mechanical ventilation and sedation, and the concomitant inactivation of the diaphragm did not result in significant changes in MyHC isoform expression (Fig. 2A) or CSA of muscle fibres expressing the type I, IIa and IIx MyHCs (Fig. 1B). At the mRNA level, however, a decreased MyHC IIx expression was observed in response to the different interventions, but this did not have any noticeable impact on the proportion of MyHC isoforms or myosin contents normalized to total protein content (Fig. 2B and 2D). This is probably due, in part, to the slow turnover rate of myosin having a half-life of more than five days [31], [32]. This is in accordance with our previous observations in a limb muscle (biceps femoris) using the same experimental model [28]. Others, on the other hand, have shown that very short periods (1–2 days) of mechanical ventilation induce a slow to fast muscle fibre transition [33] and a cell atrophy [6], [10] in the diaphragm. The animals in our study were carefully provided glucose sufficient to maintain normal blood glucose levels but calorie administration was limited to roughly 10 kcal/kg/day. While requirements for piglets in this setting are uncertain, these limited amounts did not produce measurable fiber atrophy in any animal. Based on our published [34], [35] and unpublished observations in long-term mechanically ventilated and pharmacologically paralyzed rats, it is unlikely that these discrepancies are related to species differences and variations in experimental models are more likely. In fact, experimental procedures and durations may represent key elements when considering differences between studies. For instance, one may speculate that short-term effects may only be transient. Further experiments are required to justify such conjecture.

In spite of a maintained diaphragm muscle fibre size after five days of mechanical ventilation and sedation, the maximum force decreased dramatically in membrane-permeabilized fibres expressing the type I, IIa and IIx MyHCs (Fig. 1C). The decreased specific force (maximum force normalized to fibre CSA) in response to five days of mechanical ventilation undoubtedly accounts for the impaired *in vivo* respiratory muscle function observed in response to mechanical ventilation in the porcine ICU model [4]. Specific force is mainly determined by the number of strongly attached cross-bridges and the force produced by each cross-bridge. The large loss in fibre stiffness (Fig. 1D) after five days of mechanical ventilation suggests a predominant decline in the number of myosin-actin interactions rather than a reduction in the force per interaction. This decreased number of strongly attached cross-bridges is not related to a dysfunction in the recruitment of myosin heads binding to actin (Fig. 1E) but to a reduction in the number of functional myosin molecules. The maintained myosin and actin contents normalized to total protein content (Fig. 2B) and total protein over cell volume (Fig. 2C) demonstrate that the absolute content of contractile elements is not affected by the intervention. Post-translational modifications of contractile proteins resulting in an increased number of “non-functional” myosin or actin molecules are therefore forwarded as a probable mechanism underlying the decreased specific force in response to mechanical ventilation and sedation. In line with this statement, previous studies show a significant increase in myosin and actin carbonylation in response to 1–2 days of mechanical ventilation [26]. However, five days of mechanical ventilation and sedation did not result in an increased amount of carbonyl derivates. On the contrary, a decreased amount of C = O in actin molecules was observed for piglets exposed to mechanical ventilation for five days with or without CS, NMBA and sepsis (Fig. 3), but this modification may also negatively affect myosin-actin interactions and subsequent force production [36]. At the moment we can only speculate whether this decrease in carbonyl derivates reflects a general decrease in reactive oxygen species within the diaphragm or whether related reactive radicals have a privileged role and are increased. Furthermore, other post-translational modifications may account for the decreased specific force.

### Sepsis, CS and NMBA effects

In contrast to our original hypothesis, the underlying systemic illness, sepsis, and the common components of ICU treatment, systemic administration of CS and NMBA, did not add to the negative effects of the ventilator-induced diaphragm dysfunction. In various spontaneously breathing animal models, endotoxin-induced sepsis induces an insufficient ventilation due to a decrease in diaphragm single muscle fibre specific force [15]. The massive endotoxin-induced release of pro-inflammatory cytokines, such as TNF-α and interleukins, and the increased levels of reactive oxygen species may all promote degradation and/or post-translational modifications of contractile proteins involved in strong cross-bridge formation [37]. To our surprise, in this model of controlled ventilation with unloaded diaphragm activity, we did not observe any signs of a further sepsis-induced impairment of diaphragm structure and function at the muscle cell, myofibrillar protein and mRNA levels. However, a similar finding was observed in an experimental rat model [38]. Although the exact mechanisms remain unknown, the significant differences in diaphragm muscle blood flow during mechanical ventilation and in spontaneously breathing septic animals are forwarded as one potential underlying mechanism. That is, there is a decrease in diaphragm muscle blood flow during mechanical ventilation while there is a significant increase in spontaneously breathing animals with sepsis [39]. Thus, the decrease in diaphragm blood flow may reduce exposure to sepsis-induced cytokines, reactive oxygen species, blood-borne mediators and related products known to affect muscle structure and function [38].

Long-term administration of large systemic doses of CS typically leads to a generalized steroid myopathy and an impaired ventilatory performance coupled to a decreased diaphragm muscle force production in spontaneously breathing mammals, including humans [16]. Indeed, CS induces an increased proteolysis and decreased protein synthesis rate of contractile proteins, especially myosin, resulting in a faster protein turnover rate [40], [41] and cell atrophy. In the current experiment, hydrocortisone at lower doses (5–6 mg kg^-1^ hydrocortisone/day) more like current clinical treatment strategies, did not add any deleterious effects to the inactive diaphragm, supporting recent data in mechanically ventilated rabbits [42]. Sassoon and Caiozzo [42] suggest that common molecular signaling pathways are shared by CS and mechanical ventilation. It is tempting to speculate that it may also be the case for sepsis and mechanical ventilation or NMBA and mechanical ventilation. More experiments are needed to verify such statement. With regard to NMBA, this appears highly likely since mechanical ventilation in sedated animals inactivates the diaphragm muscle in a similar way as in response to pharmacological block of neuromuscular transmission. This is in accordance with data from our group [14], [35] but differs from another study also using rocuronium [43]. The difference may be related to the duration of the experiment (<1 day vs. 5 days) and/or to the effective dose used.

### Model advantages and limitations

The porcine model offers serious advantages, including the option to follow single animals longitudinally for a relatively long period, but also presents some disadvantages, such as logistic problems, very high costs, and intrinsic limitations that restrict the duration of the experiments as well as the number of animals able to be studied. For instance, risk of under- and overfeeding could result in changes in muscle structure and function. As we found little change in morphology or protein content in all animals, it appears unlikely that the functional changes resulted from nutritional therapy. Otherwise, maintaining lung volume and consistent lung mechanics over time while avoiding high airway pressures and the risk of iatrogenic lung injury was very challenging. Measured lung compliance, tidal volumes and pressures indicate that this was possible, excepting transient periods following endotoxin infusions. Finally, the small number of animals in each group implies a risk that the study was underpowered to detect small but significant changes, as is often the case in large animal studies.

In conclusion, five days of mechanical ventilation and sedation in the experimental porcine ICU model had significant negative effects on diaphragm muscle fibre contractility and post-translational modifications of contractile proteins are forwarded as the most likely mechanism. Endotoxin-induced sepsis, corticosteroid and neuromuscular blockade did not have any additional negative effects. This was an unexpected finding that needs further scientific attention.
